# Genistein induces apoptosis of colon cancer cells by reversal of epithelial-to-mesenchymal via a Notch1/NF-κB/slug/E-cadherin pathway

**DOI:** 10.1186/s12885-017-3829-9

**Published:** 2017-12-04

**Authors:** Panpan Zhou, Chunling Wang, Zebin Hu, Wenruo Chen, Wentao Qi, Aike Li

**Affiliations:** 1 Cereals & Oils Nutrition Research Group, Academy of State Administration of Grain (ASAG), No.11 Baiwanzhuang Street, Beijing, 100037 People’s Republic of China; 2 Key Laboratory of Food Safety and Sanitation, Ministry of Education, College of Food Engineering and Biotechnology, Tianjin University of Science and Technology, Tianjin, People’s Republic of China; 3 Institue for In Vitro Diagnostic Reagents Control, the National Institutes for food and drug Control (NIFDC), Beijing, 100050 People’s Republic of China

**Keywords:** Genistein, Colon cancer cell, Apoptosis, Epithelial mesenchymal transition

## Abstract

**Background:**

Genistein has been known to inhibit proliferation and induce apoptosis in several kinds of cancer cells. While knowledge of genistein in regulating epithelial mesenchymal transition (EMT) of colon cancer cells is unknown.

**Methods:**

To investigate the effects and mechanisms of genistein on EMT of colon cancer cells, HT-29 cells were used and treated by genistein and TNF-α in this paper. EMT was determined by cell invasion assays using a transwell chamber and the expression changes of EMT-related markers were confirmed by RT–PCR, Western blotting, and immunofluorescence staining.

**Results:**

Genistein inhibited cell migration at 200 μmol/L. Genistein reversed the EMT of colon cancer cells by upregulation of E-cadherin and downregulation of N-cadherin, accompanied by the suppression of EMT related makers, such as Snail2/slug, ZEB1, ZEB2, FOXC1, FOXC2 and TWIST1. Moreover, genistein can inhibit the expression of notch-1, p-NF-κB and NF-κB, while promote the expression of Bax/Bcl-2 and caspase-3 in HT-29 cells.

**Conclusion:**

The present study demonstrated that genistein suppressed the migration of colon cancer cells by reversal the EMT via suppressing the Notch1/NF-κB/slug/E-cadherin pathway. Genistein may be developed as a potential antimetastasis agent to colon cancer.

## Background

Colon cancer, a deadly disease, is the third most common cancer type in males, and the second most common cancer type in females, with a global incidence of 1,360,000 cases and 694,000 deaths in 2012 [1]. It may be caused by many risk factors such as social environment, lifestyle especially eating habits, lack of physical activity, genetic factors etc. [2, 3]. Genistein (GEN), a potential cancer chemopreventive agent, is one of the active ingredients of soy isoflavones and has been reported to possess various biological activities, such as anti-tumor, antibacterial, lipid-lowering, estrogen-like effect [4–7]. In vitro data has shown that GEN can inhibit the growth of several colon cancer cells [8], while its particular effects on cancer cells and the mechanisms involved remain unknown [9, 10].

Epithelial mesenchymal transition (EMT) is an important process during tumor progression which affects critical steps of morphogenesis by interconverting epithelial cell types into cells with mesenchymal attributes [11]. Tumor necrosis factor-α (TNF-α) has been considered stimulated the EMT in several kinds of cancer cells which is a function that contrasts with its more established role in inducing apoptosis [7, 12, 13]. When EMT was happened, the expression of E-cadherin was found decreased, while N-cadherin, vimentin and other interstitial markers were increased, at the same time, EMT-associated transcription factor, such as Snail, Slug, ZEB1/2, Twist1/2 were upregulated [13–15].

Increasing evidence emphasizes a critical role of EMT endowing the incipient cancer cell with invasive and metastatic properties [16]. Apoptosis, which is a major way of programmed cell death, has been known to all plays an important role in the regulation of tissue development and homeostasis [17]. In recent years, the role of EMT in cell apoptosis has received considerable attention [18, 19]. It is considered that the induction of apoptotic cell death and reversal of EMT are promising emerging strategy for prevention and treatment of cancer [20, 21].

Genistein was found can induce the reversal of EMT in prostate cancer cells by an upregulated expression of epithelial marker E-cadherin and the loss of expression of mesenchymal marker vimentin [22]. GEN was also suggested can inhibit cell migration and invasion in both AsPC-1 and Notch-1-over-expressed AsPC-1 cells as Notch-1 could play a key role in the regulation of EMT [23]. However, current knowledge of GEN in regulating EMT of colon cancer cells is limited, and more detailed investigations of its function and mechanism are required.

Our previous study has proved GEN inhibits EGF-induced proliferation in colon cancer cells by promoting FOXO3 activity, targeting upstream the PI3K/Akt pathway [3]. In this study, we demonstrated that GEN can inhibite proliferation and induce apoptosis of colon cancer cells by reversal of EMT via a Notch1/NF-κB/Slug/E-cadherin pathway. This study demonstrates a new anti-tumor mechanism of genistein mediated by inhibiting the process of EMT in colon cancer cells.

## Methods

### Cell culture

HT-29 (ATCC number: HTB-38) colon cancer cells (ATCC (American Type Culture Collection), Manassas, VA) were cultured in RPMI-1640 medium (GIBCO) containing 10% FBS (Gibco), 100 U/mL penicillin and 100 U/mL streptomycin, at 37 °C and 5% CO2.

### Treatment

To examine the effects of GEN on proliferation, cells were loaded on 96-well plates for overnight and then changed to medium contained with 25–400 μmol/L GEN (LC Laboratories, Woburn, MA) respectively for another 48 h. To examine the effects of GEN on EMT, overnight monolayers were treated with medium added by GEN (200 μmol/L) and TNF-α (10 ng/mL) (Sigma-Aldrich) respectively for another 48 h. During the treatment, cells were placed in serum-free and antibiotic-free medium.

### Cell proliferation

An inhibitory effect of GEN on proliferation of colon cancer cell lines was evaluated by the MTT (3-(4,5-dimethylthiazol-2-yl)-2,5- diphenyl tetrazolium) assay. HT-29 cells were plated in 96-well plates (5000 cells per well). After incubation for 24 h, various concentrations of GEN were added into each well and each concentration was repeated in five wells. After 48 h incubation, the medium was aspirated and 0.5 μg/mL MTT was added. Cells were incubated at 37 °C for another 4 h and the formazan product was solubilized with dimethylsulfoxide (DMSO). The optical density (OD) of each well was then measured at 570 nm on an enzyme linked immunosorbent assay (ELISA) microplate reader (Multiskan EX, Labsystems, Helsinki, Finland). Each test was performed in triplicate experiments.

### Flow cytometry analysis

HT-29 cells were seeded in a 6-well plate and treated with 200 μmol/L GEN for 48 h, then cells were collected and washed with cold PBS, After fixing by ethanol (70%, *v*/v). Cells were dissolved in PBS (containing PI, RNase, EDTA and Triton X-100, pH 7.4) and incubated at 37 °C for 30 min, followed by incubation at 4 °C for 1 h in the dark. Finally, the samples were detected with a flow cytometry (Becton, Dickinson, USA).

### DAPI staining

The levels of nuclear condensation and fragmentation were observed by means of nucleic acid staining with DAPI (4′,6-diamidino-2-phenylindole) (Solarbio, Beijing, China). Briefly, HT-29 cells were plated in 6-well plates (10^5^ cells per well). After treatment, the cells were washed twice with PBS, and were fixed with methanol (MeOH), acetic acid (HAc) (3:1, *v*/v) for 10 min at 4 °C. Cells were stained with DAPI (10 mg/mL) for 20 min in the dark, and were then observed under a fluorescence microscope (Olympus BX41, Japan) in less than 15 min.

### AO/EB staining

Acridine orange and ethidium bromide (AO/EB) staining (Solarbio, Beijing, China) was carried out to further prove the cell apoptosis. Briefly, HT-29 cells were plated in 6-well plates (10^5^ cells per well). After treatment, Cells were washed with PBS for three times and then stained with the staining solution containing 100 μg/mL acridine orange and 100 μg/mL ethidium bromide for 20 min at room temperature in the dark. Cells were observed under an inverted fluorescence microscope (Olympus BX41, Japan) after the staining.

### Cell invasion assays using a transwell chamber

Cell invasion assays were performed using a Transwell chamber (8.0 μm, Polycarbonate, Corning, USA). Transwell chambers were precoated with Matrigel (1: 8; BD, Bedford, MA, USA) and exposed to ultraviolet light for 2 h following air-drying at 4 °C. Transwell chambers were then inserted into a 24-well plate containing culture medium with 20% FBS in lower chamber. Cells were starved overnight and then seeded on the upper chamber (1 × 10^5^ cells per well in 0.4% FBS culture medium). After incubation for 24 h, the filter inserts were removed from the wells and the cells on the upper side of the filter were removed using cotton swabs. Cells invaded to the underside of the filter were first fixed with methanol (15 min), and then stained with 2% ethanol containing 0.1% crystal violet powder (15 min). After being dried, the stained cells were enumerated under a microscope (Olympus BX41, Japan).

### Immunofluorescence imaging of E-cadherin

Briefly, the cell suspension (1 × 10^5^/mL) was inoculated on cover slips which were partitioned previously into the wells of a 6-well plate. After 24 h, HT-29 cells were treated with 200 μmol/mL GEN and 10 ng/mL TNF-α respectively for 48 h. Cells were fixed with 3% formaldehyde in phosphate buffered saline (PBS, pH 7.4) for 20 min, and washed thrice with PBS. Washed cells were permeabilized using 0.2% Triton X-100 and blocked in 2% BSA in PBS. Then cells were washed thrice with PBS, and incubated with the antibody E-cadherin (dilution 1:200) with 2% BSA in PBS at 37 °C for 1 h. The resulting cells were washed thrice with PBS and incubated with fluorescein FITC- labeled polyclonal goat anti-mouse IgG antibody (dilution 1:200) at 37 °C for 1 h. Cells were stained with propidium iodide (DAPI) (Sigma) and scanned by LSCM. All images were acquired using the same intensity and photodetector gain.

### Protein extraction and immunoblot

Experimental monolayers were washed with serum free media, and then total and fractionated proteins were extracted by cell lysis buffer (Cell Signaling Technology, Danvers, MA). The lysates were centrifuged at 12,000×g for 20 min at 4 °C. Equal amounts of protein, after concentration was determined by the Bradford assay (Bio-Rad, Hercules, CA), were loaded on SDS-PAGE and transferred to nitrocellulose membranes (Bio-Rad). After blocking, specific antibodies such as Bax, caspase-3, caspase-8, Bcl-2, PI3K, Notch1, p-NF-κB, NF-κB, E-cadherin, N-cadherin and β-actin from AB clonal Biotechnology Co., Ltd. (Wuhan, China) were used to perform detection. Finally each protein was detected using an enhanced chemilumi-nescence system (GE Healthcare, USA). Blot images were digitized (Chemidoc, Bio-Rad, Milan, Italy) and the area of each band was quantified using the computerized imaging system (QuantityOne, Bio-Rad). Relative optical density (arbitrary units) was normalized for control bands in each series and for protein loading (as probed by anti-actin blots). Each test was performed in triplicate experiments.

### RT-PCR procedure

Total cellular RNA was extracted using the trizol reagent (TransGen Biotech, Beijing, China) according to manufacturer’s instructions. One microgram of total RNA was reverse transcribed at 42 °C for 50 min using a TransScript First-Strand cDNA Synthesis SuperMix according to manufacturer’s instructions (TransGen Biotech, Beijing, China). PCR was then performed using Taq (TaKaRa, Shiga, Japan) polymerase. Each amplification was performed for 35 cycles, one cycle profile consisted of denaturation at 94 °C for 30 s, annealing at 55 °C for 30 s and extension at 72 °C for 120 s. PCR products were visualized by eletrophoresis through 1.2% agarose gels and quantifed with Glyko Bandscan gel analyzing software (Glyko, Novato, CA, USA). Parallel reactions were run using human GAPDH as a control for RT–PCR. The primer sequences that used for RT-PCR of slug, twist1, zeb1, zeb2, foxc1, foxc2 [24–27] and GAPDH were shown in Table 1.

**Table 1:** Primer sequences used for RT-PCR

Name	Forward primer	Reverse primer	Product size (bp)
slug	GCTACCCAAGGCCTCTCTC	GCCCAGGGCTTCATTGTATCT	478
twist1	CAGCCACTGAAAGGAAAGGC	CCTCCTGGGTGCCTCTAGAAT	418
zeb1	TGATCTGGCCATTTTCACCTGT	GACTTGCCAGGACAGCTTGC	306
zeb2	CACAGGTATGAGTGACTTTGCC	TGGCTGTGTCATGCCATTTC	302
foxc1	ATGTTCGAGTCACAGAGGATCG	TGGTGCTGGTGAGCTGAAT	305
foxc2	CGCCCGAGAAGAAGATCACC	CGCTCTTGATCACCACCTTC	384
GAPDH	GGACTCATGGTATGAGAGCTGG	GATGGCATGGACTGTGGTCT	220

### Statistical analysis

The experiments were repeated three times and the mean values were analyzed by a two-tailed unpaired t-test. The results were expressed as mean ± SD. All statistical tests were performed with statistical analysis software (SPSS, Chicago, IL, USA). The level of *p* < 0.05 was considered to be statistically significant.

## Results

### Genistein inhibit proliferation and induce apoptosis of HT-29 cells

**Fig. 1:**
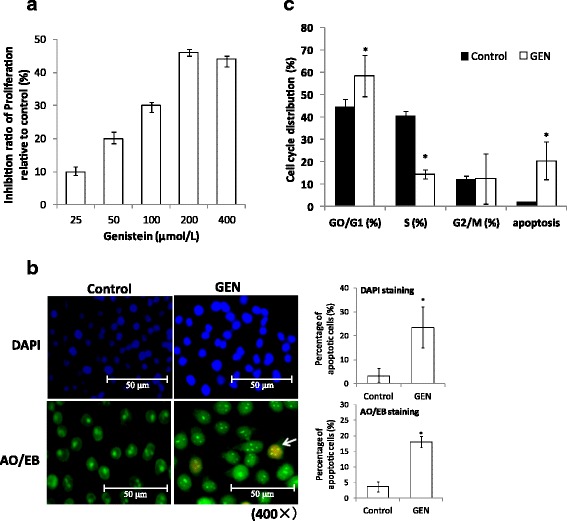
Genistein inhibit proliferation and induce apoptosis of HT-29 cells. **a** Genistein inhibited the proliferation of HT-29 cells in a dose-dependent manner. The inhibition ratio of proliferation could be up to 46 ± 1.2% at the concentration of 200 μmol/L for 48 h (*n* = 6). **b** Morphological evidence of apoptosis in HT-29 cells after 48 h of 200 μmol/L GEN treatment by DAPI and AO/EB staining. The stained nuclei were observed under a laser confocal fluorescence microscope, bar = 50 μm (×400). And the percentage of apoptotic cells per field were calculated in 3 different fields and represented by graphs (*p < 0.05, vs control). **c** Cell cycle distribution and apoptosis rate of HT-29 cells by flow cytometry after treatment with genistein (200 μmol/L) and daidzein (200 μmol/L) respectively for 48 h. (*p < 0.05, *n* = 3, vs control)

HT-29 cells were cultured with the indicated concentrations of GEN for 48 h, and cell viability was determined by MTT assay. The result showed that GEN inhibited the growth of HT-29 cells in a dose-dependent manner, with the best inhibition at 48 h in a concentration of 200 μmol/L (Fig. 1a). And the dose-dependent increase of inhibition ratio were suppressed when the concentration of GEN were over 200 μmol/L. The DAPI and AO/EB staining suggested that cell apoptosis can be significantly induced by GEN at 48 h with a concentration of 200 μmol/L (Fig. 1b). The cell-cycle phase distribution and the ratios of apoptotic cells were further determined by flow cytometry with PI staining. The percentage of cells in G1, S and G2/M phase was evaluated using Multi-cycle software, respectively. The results showed a significant increase of GEN treated cells in the G0/G1 phase from 44.60 ± 3.32% to 58.51 ± 9.20 (*p* < 0.05) compared with control, and the apoptotic rate increased significantly (p < 0.05) from 2.49 ± 0.16% to 21.50 ± 8.50% (Fig. 1c). These data indicated that GEN can induce apoptosis of HT-29 cells significantly at 200 μmol/L for 48 h. Thus, all the treatments of cells in the following experiment were carried out with 48 h of 200 μmol/L GEN.

### Genistein inhibit invasion ability of HT-29 cells

**Fig. 2:**
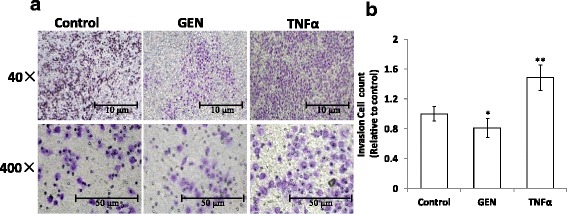
Genistein inhibit invasion ability of HT-29 cells. **a** Comparison of the cells moved into the lower chamber in each group. **b** Number of invasion cells per field were quantified in 5 different fields and represented by graphs, bar = 50 μm (*p < 0.05, **p < 0.01 vs control)

GEN was confirmed in this paper inhibit proliferation and induce apoptosis of colon cancer cells in vitro. We next characterize the effect of GEN on cell invasion in HT-29 cells by transwell chamber assay with TNF-α treatment as a positive control, since TNF-α has been proved by several research can induce EMT of kinds of cancer cells [7, 12, 28]. The results showed that few cells moved into the lower chamber of the control group, and there was fewer cells moved into the lower chamber of the GEN group, and there was significant decrease compared with control group (*n* = 3, *p* < 0.05), while the number of cells that moved into the lower chamber of the TNF-α was significantly higher than that of the control group and GEN group (*p* < 0.01). These results indicated that the invasion ability of the HT-29 cells in GEN group was significantly reduced than in the control and TNF-α group (Fig. 2a and b).

### Genistein induced reversal of EMT in HT-29 cells

**Fig. 3:**
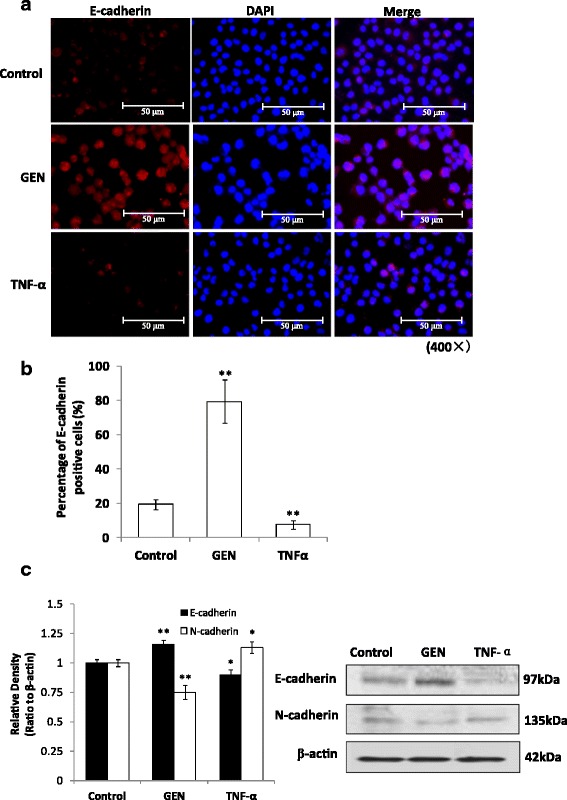
Effect of genistein and TNF-α on EMT-related markers, E/N-cadherin in HT-29 cells. **a** the protein expressions of E-cadherin in the cells treated with genistein (200 μmol/L) and TNF-α (10 ng/mL) respectively for 48 h were examined by immunofluorescence staining, bar = 50 μm (×400). **b** The percentage of E-cadherin positive cells per field were calculated in 3 different fields and represented by graphs (**p < 0.01, vs control). **c** Western blot analysis of E/N-cadherin expression in the cells treated by genistein (200 μmol/L) and TNF-α (10 ng/mL) respectively for 48 h. Density of the bands were quantified by a densitometry analysis. Data are presented after normalization by β-actin. The data shown are representative of three independent experiments. (*p < 0.05, **p < 0.01 vs control)

To further characterize the reversal of EMT induced by GEN, we analyzed the effect of GEN on EMT-related markers, E-cadherin and N-cadherin, using immunofluorescence staining and western blot assay. The Immunofluorescence results showed that the intensity of E-cadherin signal treated by GEN was obviously stronger than that of the TNF-α and control group (Fig. 3a). And the percentage of E-cadherin positive cells was 79.41 ± 12.59% in GEN group, significantly higher (*p* < 0.05) than 19.37 ± 2.94% in control group and 7.56 ± 2.50% in TNF-α group (Fig. 3b). Western-blot results further showed that TNF-α significantly reduced the E-cadherin expression (*p* < 0.05) and increased the expression of N-cadherin (*p* < 0.05) which suggested a positive effect of TNF-α on EMT (Fig. 3c). However, in parallel with the marked increase in the E-cadherin expression (*p* < 0.01), GEN significantly decreased the expression of N-cadherin (*p* < 0.01) within 48 h (Fig. 3c). These data suggested that GEN can reverse the EMT of HT-29 cells.

### Effects of genistein on the mRNA expression of invasion-related genes in HT-29 cells

**Fig. 4:**
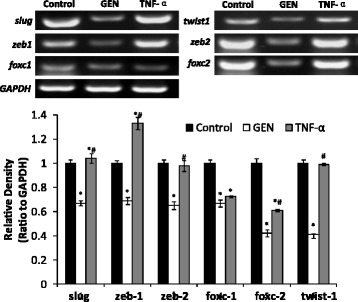
Effect of genistein and TNF-α on mRNA expression of invasion-related genes in HT-29 cells. Slug, zeb1, zeb2, foxc-1, foxc-2 and twist1 expressions in the cells treated with genistein (200 μmol/L) and TNF-α (10 ng/mL) respectively for 48 h were determined by RT-PCR analysis. Quantification of the mRNA were normalized by GAPDH. Genistein treatment leads to decreased invasion-related genes expression. The data shown are representative of three independent experiments. (*p < 0.05 vs control, ^#^ *p* < 0.01 vs GEN group)

In addition to the changes of EMT markers, the mRNA expressions of invasion-related genes in the cells were also evaluated using RT–PCR assay. The results showed that GEN significantly decreased the mRNA expression of slug, zeb1, zeb2, foxc-1, foxc-2 and twist1 in HT-29 cells (*p* < 0.05) (Fig. 4). While TNF-α significantly increased the mRNA expression of zeb1. Marked increases of the mRNA expression of slug, zeb2 and twist1 didn’t found, and fox-1 and fox-2 mRNA expression were even lower than control group. But all these mRNA expressions were significantly higher than GEN group (*p* < 0.05) (Fig. 4). These data demonstrates that GEN can significantly inhibit mRNA expression of invasion-related genes in HT-29 cells.

### Genistein inhibited the protein expression of NF-κB and p-NF-κB in HT-29 cells

**Fig. 5:**
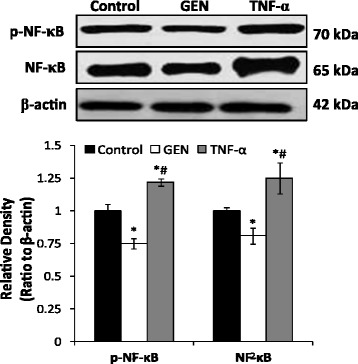
The role of NF-κB p65 in genistein induced reversal of EMT in HT-29 cells. Western blot analysis were carried out to demonstrated the of expression NF-κB p65 and phosphorylation NF-κB p65 in the cells treated by genistein (200 μmol/L) and TNF-α (10 ng/mL) respectively for 48 h. Density of the bands were quantified by a densitometry analysis. Genistein treatment leads to the decrease of both NF-κB p65 and p-NF-κB p65 expressions. Data are presented after normalization by β-actin. The data shown are representative of three independent experiments. (*p < 0.05, vs control; ^#^ *p* < 0.05, vs GEN group)

NF-κB has been found represses E-cadherin expression and enhances EMT of several kinds of cancer cells [29–31]. TNF-α can induce the EMT via the NF-κB pathway [32]. We found in this paper, GEN significantly down-regulated the expression of both NF-κB p65 and p-NF-κB p65 (*p* < 0.05) (Fig. 5). However, exposure to TNF-α resulted in remarkable increase of NF-κB p65 and p-NF-κB p65 (p < 0.05) (Fig. 5). These results suggested that GEN can reverse EMT through NF-κB pathway in HT-29 cells.

### Genistein reduce the protein expression of Notch-1 and induce the expression of Bax/Bcl-2, Caspase-8 and Caspase-3 in HT-29 cells

**Fig. 6:**
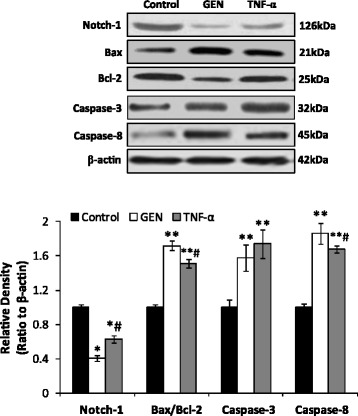
Genistein reduce the protein expression of Notch-1 and induce the expression of Bax/Bcl-2, Caspase-8. Western blot analysis were carried out to demonstrated the of expression of Notch-1, Bax, Bcl-2 and Caspase-8 in HT-29 cells treated by genistein (200 μmol/L) and TNF-α (10 ng/ml) respectively for 48 h. Density of the bands were quantified by a densitometry analysis. Data are presented after normalization by β-actin. The data shown are representative of three independent experiments. (*p < 0.05, vs control; ^#^ *p* < 0.05, vs & GEN group)

In addition, we found that GEN significantly inhibited the expression of both notch-1 (*p* < 0.05) (Fig. 6). TNF-α significantly reduced the level of notch-1 expression, however, the level was significantly higher when compared with GEN treatment (p < 0.05) (Fig. 6). It has been confirmed that the genes such as anti-apoptotic (B-cell lymphoma-2, Bcl-2) and pro-apoptotic (Bax) are important regulators of apoptosis in colon cancer cell lines [33–35]. And Caspases play a central role in apoptosis-induction [36]. Here our results showed that GEN significantly increase the expression of all the proteins including Bcl-2/Bax, Caspases-8 and Caspases-3 (*P* < 0.05) (Fig. 6). TNF-α was found also increase the expression of these proteins (p < 0.05), while the levels were lower than GEN conditions except Caspases-3 which no significant difference was found between the two treatments (Fig. 6).

## Discussion

Studies of the biological activities of GEN have always been of particular interest. Although GEN has been tested for potential anti-tumor effect, new mechanisms still are waiting for us to understand. The aim of this study was mainly to determine anti-tumor activity according the EMT. Therefore, we firstly confirmed that the exposure of HT-29 cells to GEN in a dose-dependent inhibition of cell proliferation. These results are consistent with several previous studies in HT-29 [37, 38]. Apoptosis is characterized by a series of morphological alterations such as condensation of chromatin, and fragmentation of nuclear [39]. The DAPI and AO/EB staining as well as the FCM results confirmed that GEN can induce significant apoptosis at a concentration of 200 μM for 48 h. And 200 μM was found in this study as the optimum concentration of GEN for inhibiting proliferation and inducing apoptosis.

Recently, EMT has received tremendous attention. EMT is commonly characterized by the downregulation of E-cadherin (a critical cell-to-cell adhesion molecule), and the upregulation of vimentin (a critical role in cell migration) and N-cadherin (involved in a process known as cadherin switching) [40, 41]. In our present research, in parallel with the marked increase in the E-cadherin, GEN significantly decreased the expression of N-cadherin. Immunostaining with antibodies to E-cadherin showed the changes in the localization and expression. These data suggested that GEN can reverse the level of these EMT-related proteins.

EMT is actively involved in tumor invasion and metastasis [24]. We examined the migration ability of the HT-29 cells under different treatments using a transwell chamber. The results demonstrated that the cells treated by TNF-α were more likely to metastasize than the cells treated by GEN and control. (*P* < 0.05). The cells treated by GEN even showed lower migration ability than control (*P* < 0.05). These data suggested that GEN can reverse the cells from the mesenchymal phenotype to epithelial phenotype.

TNF-α recently has been found can induce EMT in LIM 1863 cells which is a role that contrasts with its more established function in inducing apoptosis [7]. Our results here again found that TNF-α promoted EMT in HT-29 cells by downregulation of E-cadherin and upregulation of N-cadherin, accompanied by an induction of cell migration ability. These data also may confirmed previously find that TNF-α mRNA transcripts are more abundant in colorectal tumor cells than in their normal epithelial counterparts [7, 42].

Many of the EMT inducing transcription factors such as Snail1, Snail2/slug, ZEB1, ZEB2, FOXC2 and TWIST1 have been associated with tumor invasion and metastasis [24, 43]. We didn’t found significant increase of ZEB1, ZEB2, and TWIST1 mRNA expression when cells were treated by TNF-α, while the mRNA expression of slug and zeb-1 significantly increased suggested an induction of EMT by mRNA expression. The mRNA expression of FOXC1 and FOXC2 were found lower than control. This may be explained by some studies that have found the overexpression of Foxc2 enhances proliferation and inhibits apoptosis through activation of MAPK and AKT pathways in colorectal cancer [44]. On the other hand, our study clearly demonstrated that treatment by GEN decreased the mRNA expression of several mesenchymal cell markers, slug, ZEB1, ZEB2, FOXC1, FOXC2 and TWIST1 which strongly resulted in the reverse of EMT phenotype in HT-29 cells.

The family of nuclear factor-kappaB (NF-κB) transcription factors plays a pivotal role in adjusting gene transcription and governs cellular apoptosis and proliferation [45, 46]. In most normal cell, NF-κB is in an inactive form and retains in the cytoplasm which can be positively induced by TNF-α [46, 47]. NF-κB has been improved by reports can enhances EMT by repressing the expression of E-cadherin and regulation the mRNA expression of snail and zeb [29, 48]. Our data showed that GEN significantly decreased the expression of p-NF-κB and NF-κB by 25 ± 0.05% and 19 ± 0.06% respectively when compared with control (*P* < 0.05). On the opposite was the increase of p-NF-κB and NF-κB expression by 22 ± 0.04% and 25 ± 0.12% respectively compared with control under TNF-α treatment (*P* < 0.05).

Emerging evidence suggest that notch signaling pathway is an evolutionarily highly conserved mechanism for cell to cell communication and has been shown to regulate the differentiation and growth of carcinoid tumor cells [45, 49, 50]. Furthermore, over-expression of Notch-1 has been found led to the acquisition of EMT phenotype by up-regulation of mesenchymal cell markers, ZEB1, ZEB2, Snail2, and down-regulation of epithelial cell marker, E-cadherin, in pancreatic cancer cells [23]. In the present study, we demonstrated that GEN suppressed notch-1 expression significantly in HT-29 cells (P < 0.05). These data turned out that GEN can reverse EMT and induce apoptosis by impairing notch1 activation which then hindered its downstream target NF-κB p65 in HT-29 cells.

It has been confirmed that the genes such as anti-apoptotic (B-cell lymphoma-2, Bcl-2) and pro-apoptotic (Bax) are important regulators of apoptosis in colon cancer cells [33, 35]. The ratio between pro- and anti-apoptotic Bcl-2 proteins determines whether cells survive or die [51]. Bcl-2 is a target gene of NF-κB which inhibits apoptosis through interfering with caspase-8 activation [35]. Moreover, the NF-κB serves as a link between Bcl-2 expression and cell anti-apoptotic capacity [52]. In this study, the Bcl-2 was found decreased and Bax was increased as a result was the significant increased of Bax/Bcl-2 in HT-29 cells when treated by GEN. Similar results were obtained when the cells were treated by TNF-α. These data suggested a marked apoptosis induced by both GEN and TNF-α.

**Fig. 7:**
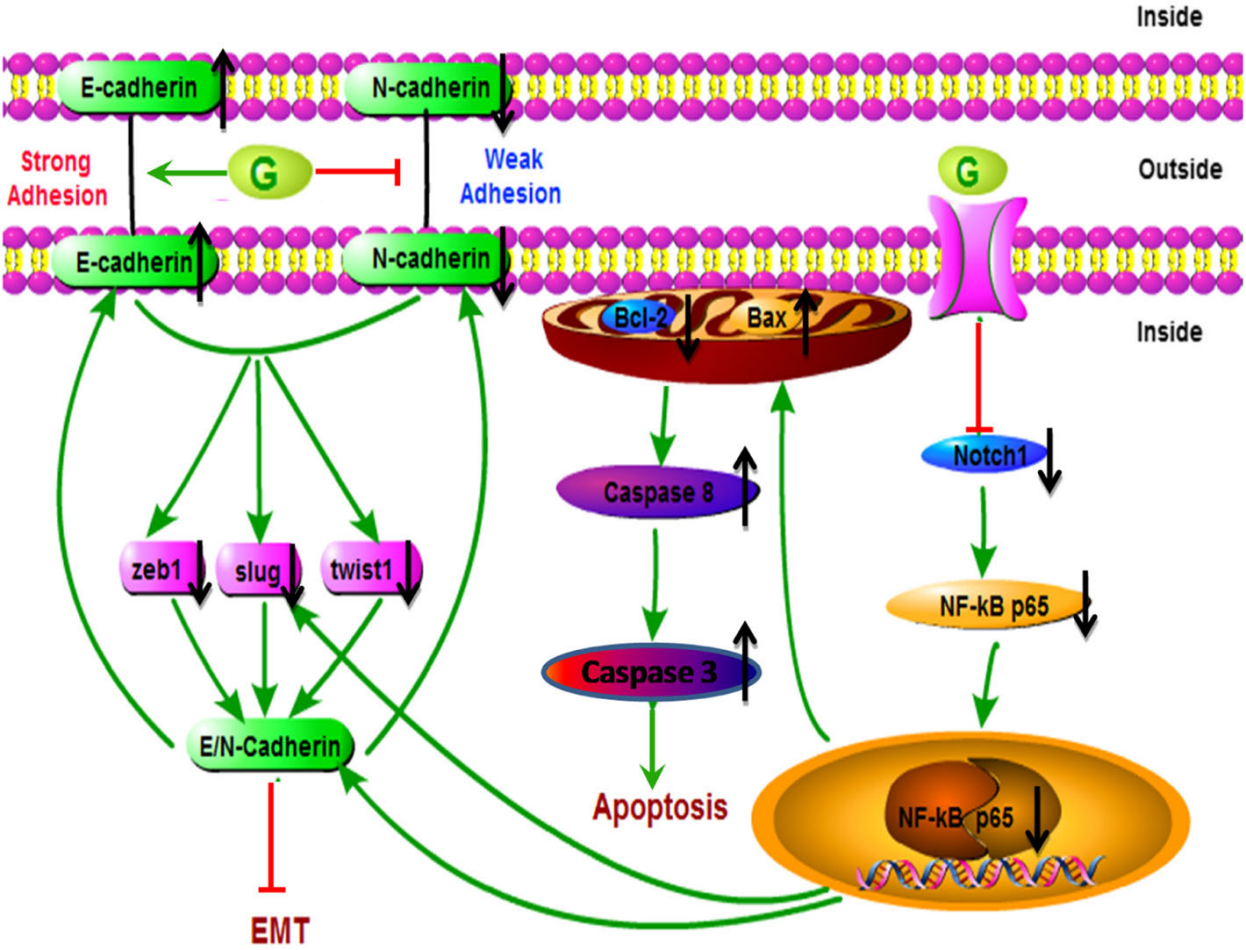
Pathways involved in apoptotic and EMT effect by genistein in HT-29 cells. Genistein reverse the EMT by promoting E-cadherin expression and inhibiting N-cadherin expression; combine with the regulations of EMT makers, Snail2/slug, ZEB1, and TWIST1. Genistein promotes Bax/Bcl-2 and caspase-8 activity by inhibiting notch-1. The notch-1 reduction leads to the inhibition of both p-NF-κB and NF-κB expression results in a reduction of EMT

Caspase-3 which can be activated by caspase-8 is a key executioner of cell apoptosis and is one of the enzymes known for the activation of different proteins that lead to programmed cell death [53]. Our results further found that the caspase-8 and caspase-3 expression was significantly increased by GEN. Taken together, our results suggest that GEN induces apoptosis of HT-29 cells via EMT and notch1 signal pathway (Fig. 7). In particular, GEN reverse the EMT by promoting E-cadherin expression and inhibiting N-cadherin expression, combine with the regulations of EMT makers such as Snail1, Snail2/slug, ZEB1, ZEB2, FOXC2 and TWIST1. Furthermore, GEN promotes Bax/Bcl-2 and caspase activity by inhibiting notch-1 pathway. And the notch-1 reduction leads to the inhibition of both p-NF-κB and NF-κB expression which again results in a negative regulation of EMT (Fig, 7).

## Conclusion

To our knowledge, no researches about the effect of GEN on EMT of colon cancer cells have been published. In this paper, we first demonstrated a novel mechanism on anticancer of GEN: the reversal of EMT. Over the years, cancer therapy had witnessed many exciting developments, but cure of cancer has still remained as complex as the disease itself. TNF-α can induce the apoptosis while with potentially induction of invasion and metastasis of colon cancer cells. GEN, however, was found by our results not only can induce the apoptosis but also can reverse the EMT of the cells. These results provide important new insights into the potential value of GEN as an anti-tumor agent.

## Abbreviations

AO/EB: Acridine orange and ethidium bromide
DAPI: 4′,6-diamidino-2-phenylindole
DMSO: Dimethylsulfoxide
ELISA: Enzyme linked immunosorbent assay
EMT: Epithelial mesenchymal transition
FCM: Flow Cytometry
GEN: Genistein
MTT: 3-(4,5-dimethylthiazol-2-yl)-2,5- diphenyl tetrazolium
NF-κB: Nuclear factor-kappaB
TNF-α: Tumor necrosis factor-α
